# Utilization of Genomic Signatures to Identify Phenotype-Specific Drugs

**DOI:** 10.1371/journal.pone.0006772

**Published:** 2009-08-28

**Authors:** Seiichi Mori, Jeffrey T. Chang, Eran R. Andrechek, Anil Potti, Joseph R. Nevins

**Affiliations:** Duke Institute for Genome Sciences & Policy, Duke University Medical Center, Durham, North Carolina, United States of America; Duke-NUS Graduate Medical School, Singapore

## Abstract

Genetic and genomic studies highlight the substantial complexity and heterogeneity of human cancers and emphasize the general lack of therapeutics that can match this complexity. With the goal of expanding opportunities for drug discovery, we describe an approach that makes use of a phenotype-based screen combined with the use of multiple cancer cell lines. In particular, we have used the NCI-60 cancer cell line panel that includes drug sensitivity measures for over 40,000 compounds assayed on 59 independent cells lines. Targets are cancer-relevant phenotypes represented as gene expression signatures that are used to identify cells within the NCI-60 panel reflecting the signature phenotype and then connect to compounds that are selectively active against those cells. As a proof-of-concept, we show that this strategy effectively identifies compounds with selectivity to the RAS or PI3K pathways. We have then extended this strategy to identify compounds that have activity towards cells exhibiting the basal phenotype of breast cancer, a clinically-important breast cancer characterized as ER-, PR-, and Her2- that lacks viable therapeutic options. One of these compounds, Simvastatin, has previously been shown to inhibit breast cancer cell growth *in vitro* and importantly, has been associated with a reduction in ER-, PR- breast cancer in a clinical study. We suggest that this approach provides a novel strategy towards identification of therapeutic agents based on clinically relevant phenotypes that can augment the conventional strategies of target-based screens.

## Introduction

Numerous advances have been achieved in the development, selection and application of chemotherapeutic agents, sometimes with remarkable clinical successes, as in the case of treatment of leukemias and lymphomas with combined cytotoxic reagents, testicular cancer with platinum, and estrogen receptor positive breast cancers with Tamoxifen [1]. Recent work has also demonstrated the value in targeting the specific molecular lesions responsible for the development and maintenance of the malignant phenotype. This is perhaps best illustrated by the example of chronic myelogenous leukemia (CML), a disease driven by the BCR-ABL oncoprotein common to virtually all patients and sensitive to Gleevec, an inhibitor of BCR-ABL [2]. Nevertheless, in the vast majority of cancers, targeted therapies are active in only a small fraction of patients [3]. An example is Herceptin, which targets breast cancers with Her2 overexpression, representing only 18–20% of all cases [4].

Conventional approaches for drug discovery have either used biochemical, target-based assays or cell-based assays that focus on a particular activity [5], [6], [7]. This continues to be an important strategy that benefits from the use of genomic studies to identify critical targets [8]. But, the same genomic technology can also be used to broaden the potential target and develop new screening methods that are grounded in relevant phenotypes. An alternative strategy might focus on a cancer-relevant phenotype rather than a specific molecular target. In fact, the past several years have seen great advances in the use of DNA microarray data to develop expression signatures that coincide with important cancer phenotypes including tumor aggressiveness, metastasis, and resistance to therapy [9], [10], [11], [12], [13], [14]. The challenge is to develop an assay system that both reflects the phenotype of interest but is also high-throughput to afford an ability to utilize large compound libraries for the identification of lead compound. We have applied a strategy based on phenotype signature to the NCI-60 drug screening dataset, taking advantage of the potential to link relevant expression signatures with action of a large number of potential cancer therapeutics. Importantly, of the 40,000 or more compounds that have been used for screening of the NCI-60 panel, a substantial number of these have been used in clinical studies. As such, a strategy that could identify therapeutics with cancer activity from amongst this group of clinically-approved agents, has the potential to rapidly move new therapeutics into clinical practice.

## Materials and Methods

### Cell culture and drug application

Methods to culture and test the drug sensitivity of 19 breast cancer cell lines are described previously [15]. We performed 12 independent *in vitro* cell proliferation assays for Simvastatin and Peplomycin, and 8 for Tamoxifen, and then calculated GI50 (growth inhibitory concentration of 50%) using GraphPad's Prism software. Averages of GI50 values were used for further statistical analysis. Simvastatin (S3449) and Tamoxifen (T5648) were purchased from LKT Laboratories and Sigma, respectively. Peplomycin was provided courtesy of Nippon Kayaku. We examined the relationship between drug response and phenotype in experiments using the non-parametric Mann Whitney U-test and linear regression using GraphPad's Prism software.

### Statistical analyses of microarray data

Analysis of expression data was described in detail previously [16], [17]. A metagene represents a group of genes that together exhibit a consistent pattern of expression in relation to an observable phenotype. Each signature summarizes its constituent genes as a single expression profile, and is here derived as the first principal component of that set of genes (the factor corresponding to the largest singular value) as determined by a singular value decomposition. Given a training set of expression vectors (of values across metagenes) representing two biological states, a binary probit regression model is estimated using Bayesian methods. Applied to the NCI-60 expression data, this leads to evaluations of predictive probabilities of each of the two states for each cell line. When predicting the pathway activation or the evidence of the phenotype of cancer cell lines, gene selection and identification is based on the training data, and then metagene values are computed using the principal components of the training data and additional cell line or tumor expression data. Bayesian fitting of binary probit regression models to the training data then permits an assessment of the relevance of the metagene signatures in within-sample classification, and estimation and uncertainty assessments for the binary regression weights mapping metagenes to probabilities of relative pathway status. Predictions of the relative status of the NCI-60 cell lines are then evaluated, producing estimated relative probabilities of the pathway activation or the evidence of the phenotype across the NCI-60 cell lines.

### Signatures for cancer cell phenotypes

To generate a signature that distinguishes basal or luminal subtype of breast cancer, we used a gene expression dataset E-TABM-157 [18] (ArrayExpress; http://www.ebi.ac.uk/arrayexpress/) that included 26 samples with basal and 25 with luminal subtypes. To validate the “basal-luminal” signature from cultured cell lines, we used three independent datasets for human *in vivo* breast cancer (GEO; http://www.ncbi.nlm.nih.gov/geo; GSE1456, GSE1561 and GSE3744) [19], [20], [21]. Gene expression signatures for RAS and PI3K used in this study were generated by adenoviral overexpression of a constitutive active mutant of H-RAS (H-RAS V12) and wild type p110α subunit of PI3K, respectively, in primary cultured human mammary epithelial cells [14], [17]. The conditions to generate the signatures are dependent upon empirically determined multiple parameters, particularly the number of genes to prioritize and the number of metagenes. These detailed conditions are described in Table S1. We also analyzed the influences of these factors to the correlations between RAS signature and the sensitivity to Hypothemycin, and between PI3K signature and the sensitivity to LY294002 (Figure S2).

### Compound sensitivity data of NCI-60 cell lines

Drug response data for NCI-60 cell lines were available from http://www.dtp.nci.nih.gov/. GI50 (drug concentration for 50% growth inhibitory effects on cells) values were available for 44,653 compounds (Release September 2005). We found the data includes many compounds that were assayed on a limited number of cell lines or whose effects did not suffice for calculating GI50s. To improve the validity of the data, we filtered the compounds that were assayed on fewer than 30 samples and also the compounds whose efficacy could not be measured at the assayed concentrations in over 50% of the assayed samples. This yielded 21,603 compounds, among which 6,638 have chemical names (or equivalents).

### Conversion of gene expression signature to drug response

We used the gene expression data of the NCI-60 on Affymetrix U133A/B chips from GEO (GSE5720) [22] for the analyses in this study. To select compounds, we calculate the Pearson correlation between the predicted probability for the phenotype of interest against the GI50 values of each of the compounds. To preserve the structure of the distribution of the phenotype probabilities and GI50 values, we calculated the *p*-values for the correlations by permuting the labels for the phenotypes 1×10^6^ times and counting the number of times we obtain the original correlation by chance. We calculated the false discovery rate (FDR) using the method of Benjamini & Hochberg to determine a cut-off value for the selection of compounds [23]. GI50 values of correlated compounds were centered and normalized by Gene Cluster 3.0 (http://bonsai.ims.u-tokyo.ac.jp/~mdehoon/software/cluster/), then visualized by Matlab, R with Bioconductor software (http://www.bioconductor.org/) or JavaTreeView (http://sourceforge.net/projects/jtreeview/).

### Mouse xenograft model

Athymic nude mice (nu/nu) were obtained from Charles River Laboratories or the Cancer Center Isolation Facility at Duke University and maintained in a sterile environment according to guidelines established by the US Department of Agriculture and the American Association for Accreditation of Laboratory Animal Care. This project was approved by the Institutional Animal Care and Utilization Committee of Duke University. Athymic mice were inoculated with in vitro propagated MDA-MB-231 cells (10^6^ in 100 µl) subcutaneously injected into each flank. Twelve days after tumor inoculation, we initiated treatment. Day 0 marks the first day of treatment. For Simvastatin treatment, tumors were randomly divided into two groups of 10 mice; control and drug treatment. Simvastatin tablets (Zocor, Merck) were mixed with food and pressed into pellets by Harlan Tekland at 1 g Simvastatin/1 kg diet to deliver a dose of 200 mg/kg mouse/day, assuming a 25 g mouse consuming 5 g chow per day. The dosage was reported to be equivalent to the maximal dosage for humans and was well tolerated by mice in a previous study [24]. Untreated animals received pellets without Simvastatin. Tumors were measured every 3 or 4 days with calipers in three dimensions. The following formula was used to calculate tumor volume: Tumor volume = WxLxHx0.5236 (W, the shortest dimension; L, the longest dimension; H, the height). The growth curves are plotted as the mean tumor volume +/− s.e.m. Average tumor size at day 0 was same between treated and control mice (Simvastatin control; 36.23 +/− 8.359 mm^3^ vs Simvastatin treatment; 35.24 +/− 5.274 mm^3^).

## Results

### Concept of an expression signature-based drug screen

To expand opportunities for cancer drug development, we have explored the concept of using cancer-relevant gene expression signatures as the basis for a screen, rather than the conventional approach of utilizing well defined biochemical targets. The logic in the use of signatures is the ability to greatly expand the number of opportunities for identifying compounds that have anti-cancer activity, recognizing the fact that a target-based approach is limited by available targets that are amenable to conventional drug screens. Pathways such as MYC and SRC represent one opportunity but the concept can go well beyond to include other, less defined, cancer relevant phenotypes that can be represented as expression signatures including poor prognosis, metastasis, or general resistance to therapies.

**Figure 1:**
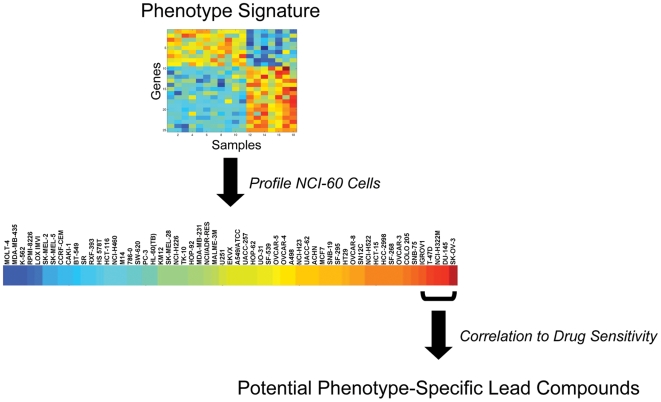
Strategy of a gene expression signature-based drug screen. A gene expression signature that reflects a clinical/biological phenotype is used to profile the NCI-60 panel to identify cells that exhibit the phenotype of interest. The predicted probability for the signature is correlated against the sensitivity to over 40,000 (21,603 after filtering) compounds to identify compounds that appear to be effective in cells exhibiting the phenotype.

To explore this concept of using signatures as the basis for a drug screening strategy, we have made use of several examples that represent potential drug targets and coupled these with data from the NCI-60 cancer cell line drug screening panel. Over 40,000 compounds have been assayed using the NCI-60 panel, thus representing a series of cell-based drug screens done in parallel [7]. Our approach uses signatures to identify cell lines within the NCI-60 panel that strongly exhibit the signature, and then identify compounds from the NCI-60 dataset that are active against those specific cell lines (Figure 1). An important aspect of the approach is the identification of multiple cell lines that exhibit a given signature and that share sensitivity to a compound or compounds.

### A screen for pathway-specific drugs

In order to illustrate the concept in the context of cell signaling pathways that are considered important cancer therapeutic targets, we have focused on two well studied pathways: RAS and PI3K. In each case, activation of these proteins and pathways is known to contribute to the development of an array of cancers [25], [26], [27]. Since each has been the subject of extensive drug development, there are many compounds identified that target components of the pathways, including compounds assayed in the NCI-60 drug screen. This then provides an opportunity to validate the concept of a signature based screen by determining if the identification of NCI-60 cell lines that exhibit a RAS or PI3K signature reveals compounds that are active against components of the respective pathways.

**Figure 2:**
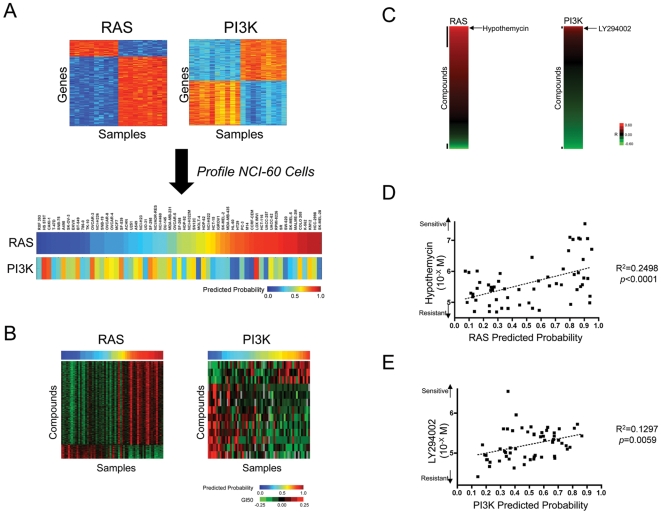
Identification of RAS or PI3K pathway-specific drugs. A. Gene expression signatures previously developed to predict RAS or PI3K pathway activation were used to predict the status of the pathways in the NCI-60 panel. The predicted probability for each oncogene activity is shown in a heatmap (lower panels; red = high and blue = low). Samples are sorted according to the RAS activity. B. A heatmap displaying the pattern of compounds correlated with RAS or PI3K pathway status. GI50s of correlated compounds with FDR less than 0.05 are shown in a heatmap (green = less sensitive and red = more sensitive) with the heatmap of predicted probabilities for RAS or PI3K activity (red = high and blue = low). Samples and compounds are sorted according to the predicted probabilities for each oncogene activity and to the correlation coefficient, respectively. RAS is positively correlated to 3616 compounds and negatively correlated to 606. For PI3K, three compounds have positive correlation and ten have negative correlation. C. Pattern of correlation of all compounds with RAS (left) or PI3K (right) predicted probability. Correlation coefficients in Pearson correlation are shown in a heatmap (green = less sensitive and red = more sensitive). Bars adjacent to the heatmap are used to indicate the compounds with FDR less than 0.05. Hypothemycin, a MEK inhibitor, is a highly correlated compound to cells with high RAS probability (rank = 331, R = 0.4998 and FDR = 0.002639). LY294002 shows strong correlation to PI3K activity without evident statistical significance (rank = 121, R = 0.3601 and FDR = 0.1463). The correlation coefficient may suggest the “strength” of the correlation. D and E. Relation between oncogenic pathway activity and pathway specific inhibitors in NCI-60 cell lines. GI50 values were plotted in the function of the predicted probalities. *P* value and R^2^ were calculated by linear regression analysis of GraphPad's Prism. D. RAS pathway and hypothemycin. E. PI3K pathway and LY294002.

We made use of previously developed expression signatures that reflect the activity of RAS and PI3K to then profile the activity of the pathways within the NCI-60 panel [14], [17] (Figure 2A). One point evident in this analysis is the distinction in cell lines exhibiting activity of the two pathway signatures. These results were then used to sort the NCI-60 cell lines based on the predictions of RAS or PI3K activity to then identify compounds that are most active against these cell lines. This result is shown in Figure 2B as a heatmap displaying activity of the compounds, sorting the cells by pathway prediction and the compounds by relative activity for a given cell line. We identified compounds as significant based on a multiple hypothesis corrected FDR (false discovery rate)<0.05. Using sequence information generated at the Sanger Institute, we find that the predicted probabilities of RAS pathway activation were indeed higher in cells with mutation of BRAF or RAS (either N-, H- or K-RAS) than the wild type cells for both loci with statistical significance [28] (RAS/BRAF wildtype vs RAS or BRAF mutant; *p* = 0.0001 by Mann Whitney test), further validating specificity of RAS prediction in NCI-60 cells.

An examination of the compounds showing a significant correlation with RAS activity revealed 3616 positively correlated compounds and 606 compounds in negative correlation. Positive correlated compounds include Hypothemycin, a drug known to target MAPK/ERK kinases (MEKs) [29] that are key downstream effectors of the RAS pathway [25], [26], [27] (rank = 331, R = 0.4998 and FDR = 0.002639). In principle, the identification of Hypothemycin in relation to RAS pathway activation is analogous to observations linking sensitivity of cells to the same MEK inhibitor based on the presence of B-RAF mutations [30] (Figure 2C and 2D). Of course, there were many additional compounds that also exhibited a positive correlation with the RAS activation phenotype, although their utility will require further experiments.

The parallel analysis using the PI3K pathway signature revealed three compounds with a positive correlation and ten with a negative correlation with FDR<0.05. The cells exhibiting the PI3K signature were largely distinct from those exhibiting the RAS signature and thus the drug profile linked to these two pathways was distinct. Indeed, there are no overlapping compounds identified in both of RAS and PI3K positive correlation. Although there were mutations in *PIK3CA* or *PTEN* in the NCI-60 cell lines, there was no correlation between these mutations and the sensitivity of LY294002, which can specifically inhibit PI3K including activity resulting from gain of function mutations [31]. Nevertheless, LY294002 is positively correlated with this PI3K signature (Figure 2C and 2E). Taken together these observations suggest an ability of the phenotype-based screen to identify relevant compounds.

### A screen for cancer phenotype-specific drugs

A second application of this approach is in a context of a phenotype that lacks a defined molecular target. An example might be a particular subgroup of cancer patients clearly at risk for disease progression but lacking currently available therapeutics. Expression data has been used to characterize human breast cancers into subtypes that reflect the cell type of origin, with a particular focus on basal and luminal subtypes. Basal type breast cancers tend to be estrogen receptor negative and exhibit a poor prognosis whereas the luminal subtype tends to be estrogen receptor positive and have a better prognosis [10]. Importantly, expression studies of cultured breast cancer cell lines have shown that these cells retain their subtype characteristics [18]. It seemed reasonable that the identification of drugs with specificity for the subtype could translate into drugs effective for the patient. This could be particularly important for the basal subtype given the general lack of effective therapeutics for this group of breast cancer patients.

**Figure 3:**
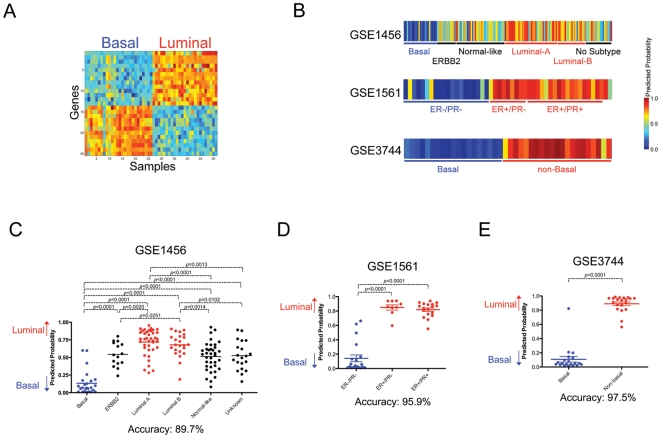
Identification of breast cancer subtype specific compounds. A. Development of a gene expression signature to distinguish basal or luminal cell type in breast cancers. Expression levels of selected genes are shown in a heatmap (high = red and low = blue). B. Validation of the “basal-luminal” signature in three independent datasets of human primary breast cancers. The predicted probability for basal (blue) or luminal (red) are shown in a heatmap with the labeling for the cell type classification by microarray (GSE1456), the immunoreactivity status for estrogen and progesterone receptor (GSE1561) or the status for basal subtype by cytokeratin expression patterns (GSE3744). C, D and E. Prediction for basal and luminal properties in *in vivo* tumor data sets. Predicted probabilities are plotted for the groups with the defined subtype and statistically evaluated using Mann-Whitney U test. A bar indicates mean value for each group. The predicted probability for basal or luminal is shown with the labeling for the cell type classification by microarray (C; GSE1456), the immunoreactivity status for estrogen and progesterone receptor (D; GSE1561) or the status for basal subtype by cytokeratin expression patterns (E; GSE3744). Accuracy of the prediction was also shown. To evaluate the accuracy, 0.5 was used as a cut-off value.

**Figure 4:**
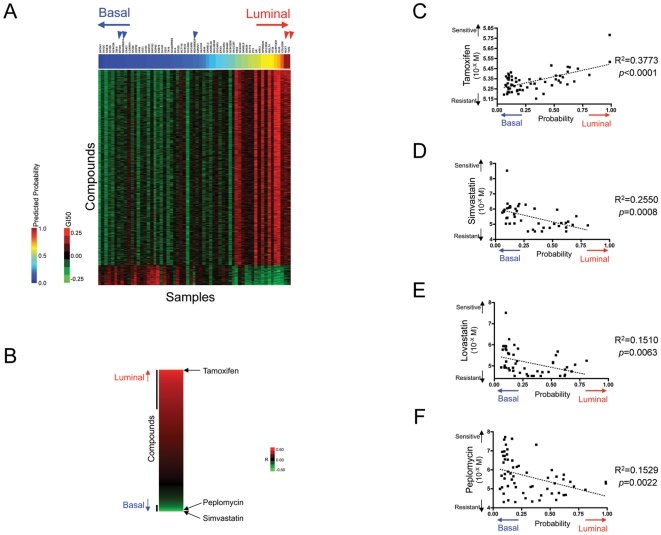
Relation between the “basal-luminal” phenotype activity and correlated drugs in NCI-60 cell lines. A. The predicted probability of NCI-60 cells for “basal-luminal” subtype and the correlated compounds. The predicted probability of NCI-60 cells for the similarity to basal (blue) or luminal (red) is shown in a heatmap and sorted according the similarity. Note that among 5 cell lines, which were characterized by the previous study [18] and are included in NCI-60 cells, every cell line was classified accurately (basal subtype; blue arrowheads; BT549, MDA-MB-231 and MDA-MB-435 and luminal subtype; red arrowheads; MCF7 and T47D). GI50 pattern for the compounds that correlated with the probability within 0.05 of FDR was shown in a heatmap (green = less sensitive and red = more sensitive). Luminal subtype correlated compounds include 5589, while 568 compounds showed correlation to basal subtype. B. Correlation pattern of all compounds with the predicted probability to “basal-luminal” signature. Correlation coefficient in Pearson correlation is shown in a heatmap (green = less sensitive and red = more sensitive). Bars adjacent to the heatmap are used to indicate FDR less than 0.05. Tamoxifen, an estrogen receptor inhibitor, is a highly correlated compound to cells with high luminal probability (rank = 57, R = 0.6140 and FDR = 0.0000). Among 568 compounds, which basal phenotype correlated within FDR of 0.05, 85 compounds have chemical names. Through Pubmed search of all 85 compounds, Simvastatin, Lovastatin and Peplomycin are found to be currently under clinical use (Simvastatin; rank = 204, R = 0.5050 and FDR = 0.006160, Lovastatin; rank = 442, R = 0.3890 and FDR = 0.02795 and Peplomycin; rank = 329, R = 0.3910 and FDR = 0.01478). Lovastatin is not shown in Figure 4B. C, D, E and F. Tamoxifen (C), Simvastatin (D), Lovastatin (E) and Peplomycin (F) and the “basal-luminal” phenotype activity. GI50 values were plotted in the function of the predicted probabilities. *P* value and R^2^ were calculated by linear regression analysis of GraphPad's Prism.

To identify compounds that potentially have activity specific for the basal phenotype, we generated a signature to distinguish basal or luminal phenotype using expression data derived from 26 basal and 25 luminal subtype cell lines grown in *in vitro* tissue culture (Figure 3A) [18]. As shown in Figure 3B–E, the signature derived from the *in vitro* model has an ability to accurately predict the status of human primary breast cancer samples from three independent datasets, validating the predictive ability of the signature. We then applied this “basal-luminal” signature to NCI-60 expression microarray data to classify the cell lines according to the degree of “basal-luminal” phenotype and identify compounds that were most active against each cell type (Figure 4A). An analysis of the significant associations revealed 5589 luminal subtype correlated compounds while 568 compounds showed a correlation to the basal subtype. Among the luminal phenotype correlated drugs, Tamoxifen was identified as a high scoring compound (Figure 4B) (rank = 57, R = 0.6140 and FDR = 0.0000). In human breast cancers, the luminal subtype is known to be largely estrogen receptor positive and sensitive to estrogen antagonists, including Tamoxifen [10]. Indeed, the predicted probability of each NCI-60 cell line for luminal subtype was strongly correlated with estrogen receptor 1 (ESR1) mRNA level (average of RMA normalized expression of 205225_at, 211233_x_at and 211234_x_at on U133A chip; R^2^ = 0.2551, *p*<0.0001 by linear regression analysis).

**Figure 5:**
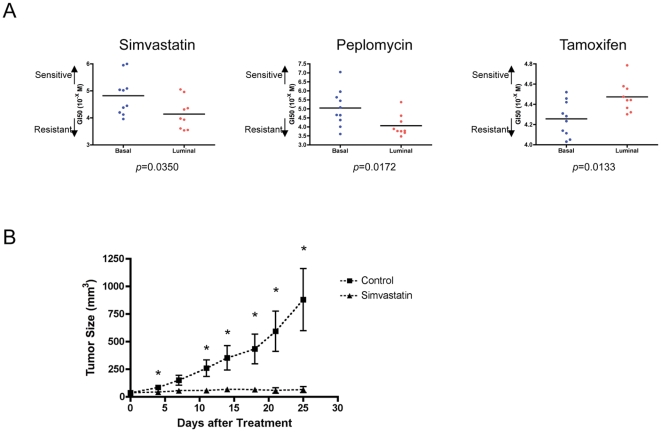
Experimental validation of compounds predicted to be active on breast cancer subtype. A. Specificity of drug sensitivity measures in breast cancer cell lines. A panel of breast cancer cell lines was classified into basal or luminal subtype based on the microarray classification (shown in Figure S1) and used for measures of sensitivity to Simvastatin (A), Peplomycin (B), and Tamoxifen (C). GI50 values were calculated after cell proliferation assays of these breast cells and averaged GI50s were plotted with *p* value (also shown in Table S3). A non-parametric Mann-Whitney U-test was used to evaluate the result statistically. B. Confirmation of *in vivo* effect of Simvastatin on a basal-type breast cancer cell line. MDA-MB-231 cells were inoculated by subcutaneous injection into mice and then the mice were treated with Simvastatin for 12 days after injections. Tumor size at day 0 was the same (see Materials and Methods in detail). Sizes of tumors were plotted as a function of days after the initiation of treatment. The unpaired t-test was used for statistical evaluation and *p* value is shown with the plot. Asterisks indicate statistically significant differences (*p* value: day 4; 0.0373, day 7; 0.0569, day 11; 0.0162, day 14; 0.0280, day 18; 0.0393, day 21; 0.0416).

Although the association of the luminal phenotype with Tamoxifen and ESR1 expression level provides an additional validation of the specificity of the methodology, a more pressing question in the context of breast cancer therapy is the basal phenotype since effective treatments for this group of patients are limited. An analysis of the compounds selected on the basis of the basal type phenotype revealed three clinically used drugs with high scores. Drugs already in clinical use are of highest priority since their characteristics, such as side effects and toxicity, have been well described. These included Simvastatin and Lovastatin, HMG-CoA reductase inhibitors, and Peplomycin, an inducer of DNA double-strand breaks [32], [33] (Figure 4B–F) (Simvastatin; rank = 204, R = 0.5050 and FDR = 0.006160, Lovastatin; rank = 442, R = 0.3890 and FDR = 0.02795 and Peplomycin; rank = 329, R = 0.3910 and FDR = 0.01478). In fact, previous studies have indicated a role for lipophilic statins such as Simvastatin and Lovastatin as inhibitors of farnesyl transferase activity and RAS/RHO activity and to have the capacity to inhibit the growth of breast cancer cells *in vitro* [34]. Based on these results, we then further tested the activity of both Simvastatin and Peplomycin in growth inhibitory assays using a panel of breast cancer cell lines. As shown in Figure 5A, each drug showed activity to those cells that exhibited the basal phenotype with a discrimination in relation to the luminal phenotype. As expected, assay of Tamoxifen using this same collection of cell lines yielded the inverse pattern, showing activity in cells exhibiting the luminal phenotype but not the basal (see also Figure S1 and Table S2 and S3).

Given the potential for Simvastatin as a basal subtype-specific drug, we evaluated the capacity of this compound to inhibit tumor growth *in vivo*, using a xenograft model with a basal subtype cell line [35]. As shown in Figure 5B, Simvastatin very effectively blocked tumor growth in this model, while the untreated controls grew rapidly. Indeed, after 25 days of treatment the tumor volume was 879.0+/−280.7 mm^3^ for untreated and 64.66+/−26.68 mm^3^ for Simvastatin treated (*p* = 0.0222 at Day 25 by unpaired t-test). Taken together, these results suggest a capacity for a signature-based screen to identify candidate drugs for new cancer therapeutics.

## Discussion

There have been major successes in the discovery and development of new cancer therapeutics based on a knowledge of the biology of the tumors, exemplified by Gleevec, Herceptin, and Tamoxifen. However, it is also true that for most of cancers, there remains a critical shortage of effective therapeutics that can match the complexity of these diseases [1], [2], [4], [36]. Many studies now provide compelling evidence that various genomic profiling approaches do have the capacity to dissect the complexity of cancers with the potential to then match drugs with patients [14], [37], [38], [39], [40]. With these advances, what becomes limiting is the availability of a sufficient repertoire of drugs that could eventually match the complexity of the cancers and thus a critical need to substantially increase the pipeline of new therapeutics to match these needs and opportunities. We believe the strategy outlined here represents one opportunity to address this need.

Although conventional drug screens have yielded many effective drugs, there are nevertheless two primary limitations that restrict the opportunity to increase the development of new drugs and for which a signature-based approach might be successful. First, many potential targets are deemed unlikely to yield drugs (not ‘druggable’), based on the biochemical properties of the protein. Oncogenic transcription factors such as MYC represent one example. MYC is known to be deregulated in a large number of human cancers yet there has been little successful development of drugs that target this important activity [6], [41], [42]. A signature reflecting MYC pathway activity could be employed to identify drugs targeting other components of the MYC pathway that might be more amenable to drug sensitivity. Indeed, various recent studies suggest that pathway activation can be linked to sensitivity to drugs targeting downstream components. As an example, cells that harbor a RAF mutation exhibit sensitivity to MEK inhibitors [30]. Also in our analysis, the identification of a MEK inhibitor based on a screen with a RAS pathway signature provides a proof-of-concept for this logic.

Second, the conventional approach that depends on the identification and detailed biochemical understanding of the nature of the target is a slow process and is limited by available understanding of cancer mechanisms [5], [6]. Once again, genomic profiles provide further opportunities for identification of relevant targets. For example, expression signatures that identify sub-classes of cancers with aggressive characteristics or cancers resistant to commonly used therapies represent therapeutic opportunities [10], [11], [12], [13], [14]. In each instance, an ability to develop therapeutics specific for these subtypes of cancer, whether breast cancer, lymphoma, or others, would be a significant step forward in expanding the arsenal of drugs that could be matched with characteristics of the individual patient. Developing a phenotype specific signature could be employed in a drug screen much like the example of the basal specific breast cancer signature shown in this work. In principle, this strategy could be expanded to virtually any relevant cancer phenotype where there is a need for further drug development, although the identification of the molecular target may be needed in order to reduce or eliminate off-target effects at further optimization step following to the initial drug discovery phase.

The number of compounds identified by the expression signatures varies considerably and may simply reflect the number of similar compounds utilized in the NCI-60 screen; indeed, there are a number of instances in which the NCI-60 compound library contains many redundant chemicals with slightly modified residues in their structures. Therefore it is not rare that the cellular response to even a substantial number of the compounds show similarity in the correlation with some molecular targets in the previous study [43] or phenotypes such as the RAS activation and luminal subtype in this study. On the other hand, only a very limited number of compounds were correlated with the PI3K signature. Nevertheless, the correlation of LY294002 with the PI3K signature, but not with *PIK3CA* or *PTEN* mutational status, suggests that the utilization of expression signatures can extend the opportunities for identifying relevant candidate drugs and can complement the previous NCI-60 drug screen methods relying on mutational information [5], [6], [7], [28], [30].

We also note that other studies have provided related strategies for drug discovery, again using expression signatures as the basis for the screen. In one instance, genes that constitute a signature are compared with genes that define response of cells to a variety of drug treatments, thus connecting drug response with a phenotype. In a second example, a signature reflecting the activity of a known cancer target becomes the actual target for drug screening [44], [45], [46], [47]. This contrasts with the approach we describe that makes use of the signature to identify cell lines that exhibit the signature and thus the phenotype of interest that can then be scored for drugs that selectively inhibit the proliferation of the cells. The principal advantage of this approach is the ability to carry out a screen where targets are not known. We do not suggest that one or the other of these strategies is better but rather suggest that they represent complementary approaches, along with conventional target-based screens, to increase opportunities for drug discovery.

Many studies now point to the fact that most cancers are extremely heterogeneous, likely reflecting a complex array of disease mechanisms. Cancers such as breast cancer are not one disease but rather a group of tens or even hundreds of diseases. As such, the likelihood that one therapeutic or even one combination of therapeutics will be effective in treating the myriad of breast cancers is very low. Rather, the complexity of the disease must be matched with an equally complex therapeutic arsenal if one hopes to effectively treat the disease. Given this, an ability to substantially increase the number of therapeutics moving through the development process is critical and we suggest that the strategy outlined could represent a key component of this process. We note that a significant advantage of this approach is the potential to identify new cancer therapeutics from a collection of drugs that have already progressed through the initial stages of drug development. As such, this greatly accelerates the process of bringing new agents to clinical use. As with any example of a drug screen, candidates identified by a signature-based screen must be evaluated for their potential for further development. This can involve a number of criteria typically used in the drug development process but including other indications for potential activity in a given context. As an example, the identification of Simvastatin as a potential breast cancer therapeutic useful for tumors of the basal subtype is interesting in light of previous work that has shown a role for statins, including Simvastatin, in breast cancer prevention [48]. Even more relevant with respect to the potential specificity for breast cancer with a basal phenotype is a recent population based study showing a reduction in ER/PR negative breast tumors in women treated with lipophilic statins such as Simvastatin [49]. The basal subtype is characterized by an ER/PR negative status suggesting that the reduction in this form of cancer could indeed reflect a selective effect on the basal subtype. Given this connection, and the fact that Simvastatin is an approved drug with known toxicity profiles, we suggest that a clinical study is warranted to evaluate the activity of Simvastatin in women with the basal subtype of disease.

## Supporting Information

Figure S1 Classification of cultured breast cancer cell lines. A. Unsupervised classification using the basal-luminal classifier genes. For the classifier, we used gene sets (305 probes) described in the original study (1). Expression values of RMA (robust multichip average) normalized data for these probes were gene-centered and normalized. We then performed hierarchical clustering by average linkage. Expression levels of selected genes are shown in a heatmap (high = red and low = blue). B. Supervised classification using a binary regression method. The predicted probability of the 19 breast cancer cells for the similarity to basal (blue) or luminal (red) is shown in a heatmap and sorted according to the similarity. Note that the HCC1468 cell line, which was classified as a luminal subtype in the original work (1), was predicted to belong to the basal subtype by both the unsupervised and supervised methods. We therefore have designated HCC1468 cells as basal phenotype for this study. Reference 1. Neve RM, Chin K, Fridlyand J, et al. A collection of breast cancer cell lines for the study of functionally distinct cancer subtypes. Cancer Cell 2006; 10: 515–27.
(10.20 MB EPS)

Figure S2 Influence of empirical parameters used for prediction on correlation with compounds. Pearson correlation analysis of the RAS prediction with Hypothemycin sensitivity (A) and that of the PI3K prediction with LY294002 sensitivity (B). We altered the number of genes to prioritize (left panels) or the number of metagenes (right panels) and predicted the status of the phenotype of the NCI-60 cell lines. We correlated the predicted probability with sensitivity data and show the correlation coefficient with the altered parameters. An arrow indicates the parameter used in this study.
(6.27 MB EPS)

Table S1 Detailed conditions for generation of cancer relevant signatures. The parameters used in this study are shown.
(0.03 MB DOC)

Table S2 Compounds correlated with basal subtype in NCI-60 data. 85 compounds with chemical names (or equivalents), which are correlated with the basal subtype, are shown in this table. Clinically used drugs are labeled by bold font. NSC numbers are IDs for each compound in NCI-60 data. Abbreviations: R; correlation coefficient and FDR; false discovery rate.
(0.14 MB DOC)

Table S3 GI50s of Simvastatin, Peplomycin and Tamoxifen in our breast cancer cell lines. GI50 values of Simvastatin, Peplomycin and Tamoxifen for our 19 breast cancer cell lines are shown with standard error of the mean. Microarray-based subtype classification is also indicated in the table.
(0.04 MB DOC)
