# Supplementary material for: Utilization of Genomic Signatures to Identify Phenotype-Specific Drugs
Source: PLoS One. 2009 Aug 28;4(8):e6772. doi: 10.1371/journal.pone.0006772 (PMC2729377; doi:10.1371/journal.pone.0006772)
Supplement: Table S1 — Detailed conditions for generation of cancer relevant signatures. The parameters used in this study are shown. (0.03 MB DOC) [file pone.0006772.s003.doc]

Table S1

| Signature | Source | # of Genes | # of Metagenes | Burn Ins | Confident Intervals | Skips | Iterations | Quantile Normalization |
| --- | --- | --- | --- | --- | --- | --- | --- | --- |
| RAS | Bild 2006 | 125 | 2 | 2000 | 95 | 2 | 2000 | + |
| PI3K | Potti 2006 | 370 | 2 | 2000 | 95 | 2 | 2000 | + |
| Basal-Luminal | Neve 2006 | 25 | 2 | 2000 | 95 | 2 | 2000 | + |
