# Supplementary material for: Utilization of Genomic Signatures to Identify Phenotype-Specific Drugs
Source: PLoS One. 2009 Aug 28;4(8):e6772. doi: 10.1371/journal.pone.0006772 (PMC2729377; doi:10.1371/journal.pone.0006772)
Supplement: Table S2 — Compounds correlated with basal subtype in NCI-60 data. 85 compounds with chemical names (or equivalents), which are correlated with the basal subtype, are shown in this table. Clinically used drugs are labeled by bold font. NSC numbers are IDs for each compound in NCI-60 data. Abbreviations: R; correlation coefficient and FDR; false discovery rate. (0.14 MB DOC) [file pone.0006772.s004.doc]

Table S2

| NSC | Rank | R | *p*-value | FDR | NAME |
| --- | --- | --- | --- | --- | --- |
| 629738 | 17 | 0.633 | 0 | 0 | 1-Naphthalenecarboxamide, N,N'-1,8-(octanediyl)bis- |
| 630375 | 21 | 0.578 | 0 | 0 | 2H-1-Benzopyran-2-one, 4-(2-benzofuranyl)-7-methoxy- |
| 619907 | 23 | 0.69 | 1.00E-06 | 0.000141196 | Mn(II) 3,5-diiodosalicylate |
| 94889 | 35 | 0.572 | 2.00E-06 | 0.000236098 | Flavone, 5-hydroxy-3',4',5',6,7,8-hexamethoxy- |
| 627666 | 37 | 0.569 | 3.00E-06 | 0.000304268 | 1H-Benzo[de]-1,3-benzodioxolo[5,6]quinoline-6a- carbonitrile, 6-benzoyl-1,2-dimethoxy- |
| 680553 | 43 | 0.562 | 5.00E-06 | 0.00043206 | 2-Quinoxalinecarboxylic acid, 3-[(4-fluorophenyl)amino]-, ethyl ester |
| 625350 | 44 | 0.56 | 5.00E-06 | 0.00043206 | Benzenepropanoic acid, .beta.-(benzoylamino)-.alpha.- hydroxy-, (9,10-dihydro-9,10-dioxo-2-anthracenyl)methyl ester (R*,R*) |
| 630684 | 55 | 0.539 | 9.00E-06 | 0.000631256 | 8-Quinolinecarboxaldehyde, 2-(2,4-dinitrophenyl)-2-methyl- hydrazone |
| 658469 | 57 | 0.551 | 1.00E-05 | 0.000648739 | 1,3-dimethyl-2,4-dioxo(1H,3H)pyrimido dimethyltetrathiafulvalene |
| 631346 | 62 | 0.609 | 1.30E-05 | 0.000761081 | Dubiusine |
| 682771 | 81 | 0.526 | 4.00E-05 | 0.001479658 | 5H-Pyrido[3,2-d][2]benzazepin-6(7H)-thione, 2-(4-chlorophenyl)-4-phenyl- |
| 648333 | 85 | 0.664 | 4.40E-05 | 0.001535593 | Scutellaprostin A |
| 681271 | 87 | 0.506 | 4.50E-05 | 0.001555416 | 1,3,6-Triphenyl-oxazolo(5,4-d)pyrimidin-2',4(1H,3H)-dion |
| 633242 | 111 | 0.511 | 7.80E-05 | 0.002064993 | Methanone, 2,6-pyridinediylbis[(4-fluorophenyl)- |
| 382029 | 115 | 0.514 | 8.40E-05 | 0.002137399 | Maxima isoflavone G |
| 674674 | 125 | 0.494 | 0.000112 | 0.002549564 | Pectenotoxin 1 |
| 254677 | 126 | 0.49 | 0.000116 | 0.002596837 | Phomazarin |
| 678125 | 130 | 0.51 | 0.000121 | 0.002645712 | 1H-indazole, 3-methoxy-1-[(2-methoxyphenyl)methyl]- 5-nitro- |
| 680721 | 134 | 0.479 | 0.000134 | 0.002805041 | 5H-1,4,8,11-Benzotetrathiacyclotridecin- 13,14-dicarbonitrile, 2, 3,6,7,9,10-hexahydro- |
| 678102 | 139 | 0.483 | 0.000164 | 0.003188922 | 5-Hydroxy-3,7-dimethoxy-3',4'-methylenedioxyflavone |
| 620327 | 141 | 0.47 | 0.000165 | 0.003199726 | Carbonimidodithioic acid, [5-(4-nitrophenyl)-1,3,4- thiadiazol-2-yl]-, dimethyl ester |
| 666707 | 143 | 0.496 | 0.000171 | 0.003243295 | 2-bishydroxyimino-4-(4-morpholinyl)-1-phenylmethyl bicyclo[2.2.2]octane |
| 338643 | 167 | 0.452 | 0.000301 | 0.004528205 | 6-Phenyl-6H-indeno[1,2-c]isoquinoline-5,11-dione |
| 622613 | 169 | 0.459 | 0.000315 | 0.004638681 | 3H-Naphtho[1,8-bc]furan-2-carboxylic acid, 4,5-dihydro-6-nitro-, ethyl ester |
| 630717 | 170 | 0.484 | 0.000322 | 0.004693769 | Nilacrone |
| 665549 | 184 | 0.452 | 4.00E-04 | 0.005294853 | Azoxydapsone {1,2-bis-[4-(4'-aminophenylsulfonylphenyl)]- diazine-1-oxide} |
| 289487 | 200 | 0.44 | 5.00E-04 | 0.006010851 | Asterriquinone |
| 633782 | 204 | 0.505 | 0.000521 | 0.006160461 | **Simvastatin** |
| 680114 | 208 | 0.443 | 0.000558 | 0.006374656 | Cayaponoside C5b |
| 682447 | 220 | 0.446 | 0.000638 | 0.006932955 | 3.beta.-Acetoxy-16-oxo-17-aza-D-homo-5-androsten-17.alpha.-one N-acetyl hydrazone |
| 678100 | 229 | 0.435 | 0.000711 | 0.007427337 | Isokanugin |
| 363072 | 239 | 0.481 | 0.000836 | 0.00815723 | [4,4'-Bipiperidine]-1,1'-dicarbothioic acid, bis[[1-(2-pyridinyl)ethylidene]hydrazide] |
| 628875 | 255 | 0.504 | 0.000967 | 0.008900767 | Rutacridone |
| 674276 | 262 | 0.42 | 0.001053 | 0.009403869 | Pyrazino[1,2-a]benzimidazole, 1,3-diphenyl- |
| 640328 | 263 | 0.515 | 0.001057 | 0.00942012 | Ternatin |
| 129364 | 270 | 0.427 | 0.001111 | 0.009677796 | [1]Benzothieno[2,3-b]quinoxaline |
| 630715 | 275 | 0.463 | 0.001213 | 0.010236109 | Acetoacrone |
| 638838 | 282 | 0.442 | 0.001286 | 0.010632016 | {2,2'-[1,2-Ethanediylbis(iminomethyl)]diphenolato} dioxouranium(VI)-hydrate |
| 674277 | 291 | 0.405 | 0.001468 | 0.01155674 | Pyrazino[1,2-a]benzimidazole, 3-(4-methylphenyl)- 1-phenyl- |
| 350895 | 297 | 0.442 | 0.001508 | 0.011731121 | Bleomycinamide, N1-[3-[(4-aminobutyl)amino]propyl]- |
| 382026 | 306 | 0.42 | 0.001675 | 0.012525104 | Maxima isoflavone A |
| 632848 | 310 | 0.444 | 0.001757 | 0.012963276 | 4-Bromo-(2'-methyl-4'-N,N-bis-2'-cyanoethylamino) azobenzene |
| 382386 | 317 | 0.446 | 0.001937 | 0.013778403 | 2,4-Imidazolidinedione, 3,3'-(1,6-hexanediyl)bis[5,5- diphenyl- |
| 625774 | 323 | 0.415 | 0.002031 | 0.014174135 | N2,N4,N6-Tribenzoyloxy-methyl-N2,N4,N6-trimethylmelamine |
| 276382 | 329 | 0.391 | 0.002179 | 0.014784214 | **Pepleomycin** |
| 651688 | 338 | 0.433 | 0.002405 | 0.01569644 | Contignasterol |
| 685704 | 345 | 0.396 | 0.002507 | 0.016075607 | 4'-methylpenduletin |
| 641261 | 356 | 0.424 | 0.002716 | 0.01690399 | Quinaldopeptin |
| 628694 | 361 | 0.39 | 0.002885 | 0.017595894 | 1H-Pyrazole-1-carboxaldehyde, 4,5-dihydro-3-(7-hydroxy- 4-methyl-2-oxo-2H-1-benzopyran-8-yl)- 5-phenyl- |
| 89850 | 368 | 0.423 | 0.003113 | 0.018470239 | ANHYDROBENZOQUINONE, BIS |
| 633001 | 372 | 0.385 | 0.003165 | 0.018635458 | Benzene, 1,1'-sulfonylbis(2-nitro- |
| 55720 | 380 | 0.423 | 0.003369 | 0.019392621 | Phosphinic acid, bis(1-aziridinyl)-, 2-naphthalenyl ester |
| 45194 | 385 | 0.401 | 0.003458 | 0.019679445 | Fat Red RS |
| 56737 | 386 | 0.407 | 0.003473 | 0.019744005 | Propionic acid, 2, 2'-thiodi-, bis[(m-methoxybenzylidene)hydrazide] |
| 294979 | 393 | 0.375 | 0.003696 | 0.0206104 | Bleomycin-BAPP |
| 59729 | 396 | 0.395 | 0.003735 | 0.020731553 | Sparsomycin |
| 610744 | 397 | 0.382 | 0.003751 | 0.020782984 | Imidazole, 1-(4-chlorophenyl)-4-(4-nitrophenyl)- |
| 675772 | 403 | 0.378 | 0.004118 | 0.022014537 | Glutamic acid, N-[4-[[6-(trifluoromethyl)-2-quinoxalinyl] amino]benzoyl]-, diethyl ester |
| 613011 | 407 | 0.375 | 0.00425 | 0.022459088 | 2-Thiazolidinone, 4-(17-hydroxy-5, 12-dimethyl-3-oxo-2,16- dioxabicyclo[13.3.1]nonadeca-4,8, 10-trien-17-yl)-, [1R-[1R*,4Z,8E,10Z,12S*,15R*,17R*(R*)]]- |
| 4702 | 408 | 0.379 | 0.004335 | 0.022757679 | 9H-Carbazole, 3-nitro-9-nitroso- |
| 657991 | 424 | 0.375 | 0.005063 | 0.025149687 | 4H-Pyrazolo[3,4-d][1,3]thiazin-6-amine, 1-(2-hydroxybenzoyl)-4-(4-methoxyphenyl)-3-methyl- |
| 626885 | 425 | 0.36 | 0.005142 | 0.02541355 | Benzenepropenoic acid, 3-methoxy-4-[(phenylmethoxy) carbonyl]-, 4-(acetylamino)phenyl ester |
| 618857 | 438 | 0.359 | 0.005637 | 0.027013334 | 4-Piperidinone, 3,5-bis[[(4-methylthio)phenyl]methylene]-, hydrochloride |
| 633781 | 442 | 0.389 | 0.005931 | 0.027957101 | **Lovastatin** |
| 7520 | 447 | 0.356 | 0.006349 | 0.029282119 | Jervine |
| 623794 | 450 | 0.382 | 0.006392 | 0.029386332 | 1,4-Benzodioxin-2-carboxamide, 6-(4-oxo-4-H-1- benzopyran-2-yl)-N-(3-pyridinylmethyl)- |
| 653846 | 479 | 0.39 | 0.008206 | 0.034972227 | Benzoic acid, 2-hydroxy-, 2-[(3,4-dimethoxyphenyl)(cyano) methyl]hyrazide |
| 633555 | 483 | 0.366 | 0.008482 | 0.035725609 | Acylfulvene |
| 613575 | 492 | 0.364 | 0.009075 | 0.0372997 | CDPI-trimer methyl ester |
| 624953 | 496 | 0.351 | 0.009216 | 0.03767851 | Allyl 2,3,4-tri-O-benzyl-6-O-(tert-butyldimethylsilyl)- .alpha.-D-glucopyranoside |
| 96349 | 500 | 0.345 | 0.009552 | 0.03855286 | Dibenzo[a,g]quinolizinium, 2,3,10,11-tetramethoxy-8-methyl-, chloride |
| 9170 | 506 | 0.341 | 0.010041 | 0.039947647 | Colchicine, N-deacetyl-10-thio- |
| 639600 | 507 | 0.375 | 0.010055 | 0.039966544 | Tetrakis(1,3-diphenylpropane-1,3-dionato)-.mu.-bis (ethylen-1, 2-diethyl-1,2-diphenolato-O1,O2)dititanium(IV) |
| 640335 | 512 | 0.332 | 0.010201 | 0.040316905 | 2-Propen-1-one, 1-[2-[(3,4-dichlorophenyl)amino]- 4-methyl-5-thiazolyl]-3-(5-benzodioxolyl)-, (2, 4-nitrophenyl)hydrazone |
| 638381 | 514 | 0.371 | 0.010316 | 0.040593178 | Bis[(1-phenylbutane-1,3-dionato)distearato]titanium(IV) |
| 305222 | 518 | 0.333 | 0.010612 | 0.041321384 | Cytochalasin H |
| 633253 | 524 | 0.333 | 0.010999 | 0.042257051 | 4,8-Ethenobenzo[1,2-c:4,5-c']dipyrrole-1,3,5,7(2H,6H)- tetrone, 2, 6-bis(3-ethynylphenyl)-3a,4,4a,7a,8,8a- hexahydro- |
| 682769 | 527 | 0.333 | 0.011284 | 0.043004032 | 5H-Pyrido[3,2-d][2]benzazepin-6(7H)-one, 4-(3, 4-dimethoxyphenyl)-2-phenyl- |
| 376128 | 541 | 0.333 | 0.012044 | 0.044871208 | Dolastatin-10 |
| 658396 | 550 | 0.322 | 0.012743 | 0.046638765 | N-(3-Aziridinylpropyl)-1,4-diaminobutane |
| 150817 | 553 | 0.32 | 0.013029 | 0.047305124 | Nysert |
| 657992 | 560 | 0.323 | 0.013369 | 0.048026623 | 4H-Pyrazolo[3,4-d][1,3]thiazin-6-amine, 1-(2- hydroxybenzoyl)-3-methyl-4-(4-nitrophenyl)- |
| 638850 | 563 | 0.322 | 0.013497 | 0.048306112 | UCN-01 |
| 627727 | 565 | 0.36 | 0.013757 | 0.048948299 | .beta.-Amanitin |
| 645429 | 567 | 0.343 | 0.013863 | 0.049200327 | Pyrrolo[3,2,1-hi]indole-7-carboxylic acid, 1-ethyl-1,2,4,5,5a,6, 8a,8b-octahydro-5a-phenyl-, methyl ester |
