# Supplementary material for: Utilization of Genomic Signatures to Identify Phenotype-Specific Drugs
Source: PLoS One. 2009 Aug 28;4(8):e6772. doi: 10.1371/journal.pone.0006772 (PMC2729377; doi:10.1371/journal.pone.0006772)
Supplement: Table S3 — GI50s of Simvastatin, Peplomycin and Tamoxifen in our breast cancer cell lines. GI50 values of Simvastatin, Peplomycin and Tamoxifen for our 19 breast cancer cell lines are shown with standard error of the mean. Microarray-based subtype classification is also indicated in the table. (0.04 MB DOC) [file pone.0006772.s005.doc]

Table S3

| **Cells** | **Subtype** | **Simvastatin** | **Peplomycin** | **Tamoxifen** |
| --- | --- | --- | --- | --- |
| BT20 | Basal | 4.445 +/- 0.053 | 5.076 +/- 0.059 | 4.309 +/- 0.006 |
| BT549 | Basal | 5.019 +/- 0.048 | 5.952 +/- 0.101 | 4.139 +/- 0.014 |
| HCC1143 | Basal | 4.121 +/- 0.018 | 4.380 +/- 0.038 | 4.283 +/- 0.009 |
| HCC1428 | Basal | 4.378 +/- 0.094 | 5.450 +/- 0.062 | 4.457 +/- 0.046 |
| HCC1806 | Basal | 4.198 +/- 0.019 | 7.014 +/- 0.154 | 4.519 +/- 0.040 |
| HCC38 | Basal | 3.957 +/- 0.027 | 5.638 +/- 0.042 | 4.113 +/- 0.079 |
| Hs578T | Basal | 5.086 +/- 0.063 | 4.006 +/- 0.094 | 4.051 +/- 0.087 |
| MDA-MB-157 | Basal | 5.039 +/- 0.023 | 4.659 +/- 0.071 | 4.233 +/- 0.005 |
| MDA-MB-231 | Basal | 5.997 +/- 0.026 | 3.602 +/- 0.014 | 4.030 +/- 0.082 |
| MDA-MB-435s | Basal | 5.948 +/- 0.020 | 4.648 +/- 0.060 | 4.421 +/- 0.032 |
| BT474 | Luminal | 4.356 +/- 0.041 | 3.765 +/- 0.039 | 4.578 +/- 0.088 |
| BT483 | Luminal | 3.551 +/- 0.036 | 3.778 +/- 0.074 | 4.439 +/- 0.031 |
| CAMA1 | Luminal | 4.309 +/- 0.052 | 3.773 +/- 0.078 | 4.364 +/- 0.012 |
| MCF7 | Luminal | 3.975 +/- 0.015 | 3.471 +/- 0.062 | 4.301 +/- 0.002 |
| MDA-MB-361 | Luminal | 3.605 +/- 0.058 | 3.754 +/- 0.040 | 4.321 +/- 0.006 |
| MDA-MB-453 | Luminal | 4.960 +/- 0.065 | 4.296 +/- 0.061 | 4.785 +/- 0.043 |
| SKBR3 | Luminal | 5.052 +/- 0.085 | 4.618 +/- 0.068 | 4.442 +/- 0.035 |
| T47D | Luminal | 3.988 +/- 0.030 | 5.371 +/- 0.111 | 4.554 +/- 0.046 |
| ZR75 | Luminal | 3.537 +/- 0.034 | 3.667 +/- 0.016 | 4.475 +/- 0.044 |
